# Estimating the evolutionary fitness of specific synonymous codon changes

**DOI:** 10.1093/molbev/msag199

**Published:** 2026-08-11

**Authors:** Vitor A C Pavinato, Jody Hey

**Affiliations:** Department of Biology, Temple University, Philadelphia, PA 19122, USA; Department of Biology, Temple University, Philadelphia, PA 19122, USA

**Keywords:** synonymous codon changes, polymorphism frequencies, selection-mutation-drift model, codon usage, weak selection, mRNA structure

## Abstract

Synonymous mutations do not alter proteins but undergo natural selection in many species. For *Drosophila melanogaster*, reports on selection strength vary from undetectable to surprisingly strong. Here we apply a new method to estimate the population selection coefficient (2Ns) for all 134 ordered pairs of synonymous codon changes. The method uses ratios of site frequency spectra for synonymous codon changes and matched neutral changes, avoiding reliance on divergence data or codon frequencies and remaining relatively insensitive to demographic history. Results indicate that natural selection on synonymous codons is weak, with |2Ns|<2.30 for all pairs of codons and |2Ns|<1 for 49% of codon changes. Despite being derived solely from polymorphism data, codon fitness estimates are strongly correlated with observed codon frequencies. A selection-mutation-drift model based on these estimates accurately predicts codon usage, while a model based on mutation alone fails. Codon fitness estimates also predict expression-associated codon usage, with higher-fitness codons used more often in highly expressed genes. However, the effect of expression on selection strength is modest, with a 10-fold increase in expression predicted to increase the average strength of selection on synonymous codons by only 14%. Finally, we detect clear signs that selection favors codon changes that stabilize mRNA secondary structure. The convergence of multiple independent lines of evidence validates this polymorphism-based approach and provides a coherent framework for understanding selection on synonymous site evolution.

Significance statement Synonymous mutations do not change proteins yet affect gene expression in multiple ways. Here we provide the first fitness estimates for each type of synonymous codon change in *Drosophila*. Using only allele frequencies—without divergence data, codon counts, or assumptions about preferred codons—we estimate the fitness of all 134 single-step synonymous codon changes in *Drosophila melanogaster*. Results reveal weak but non-negligible selection, with codon fitness values accurately predicting codon usage patterns, including expression-dependent biases, codon covariation across genes, and mRNA secondary structure effects. This work provides fitness estimates for individual synonymous mutations, providing a direct population-level view of how subtle genetic changes shape molecular evolution and establishing a general strategy for quantifying weak selection using polymorphism data alone.

## Introduction

Although they do not alter proteins, synonymous mutations are not selectively neutral in many species. The evidence for this comes from multiple sources, including experimental evolution studies (Bailey et al. 2021), a wide array of correlations with diverse aspects of gene expression (Plotkin and Kudla 2011), phylogenetic studies (McVean and Vieira 2001; Singh et al. 2007), and population genetic analysis (Lawrie et al. 2013). As expected from theory, the evidence tends to be strongest in species that are thought or known to have large effective population sizes (Hershberg and Petrov 2008).

Synonymous mutations offer a unique opportunity to study natural selection because some aspects of their impact on gene expression are partly understood, and because they provide a form of replication that other classes of variants do not offer. Synonymous mutations of a given codon-to-codon class (e.g. CCC→CCT) are expected to influence gene expression in similar ways through a shared set of molecular mechanisms affecting gene expression (Liu et al. 2021). By this commonality, we mean that all mutations in the same ordered codon change class cause the same change in the base that is transcribed, the same change in codon–anticodon pairing, the same change in GC content, and are probably similar in many other ways associated with gene expression. This commonality is only approximate, as local effects can arise from gene regulatory differences, mRNA secondary structure, variation in local tRNA pools, and other contextual factors. However, there are no comparably crisp systems for classifying nonsynonymous or regulatory mutations by shared mechanisms or expected effect.

**Figure 1:**
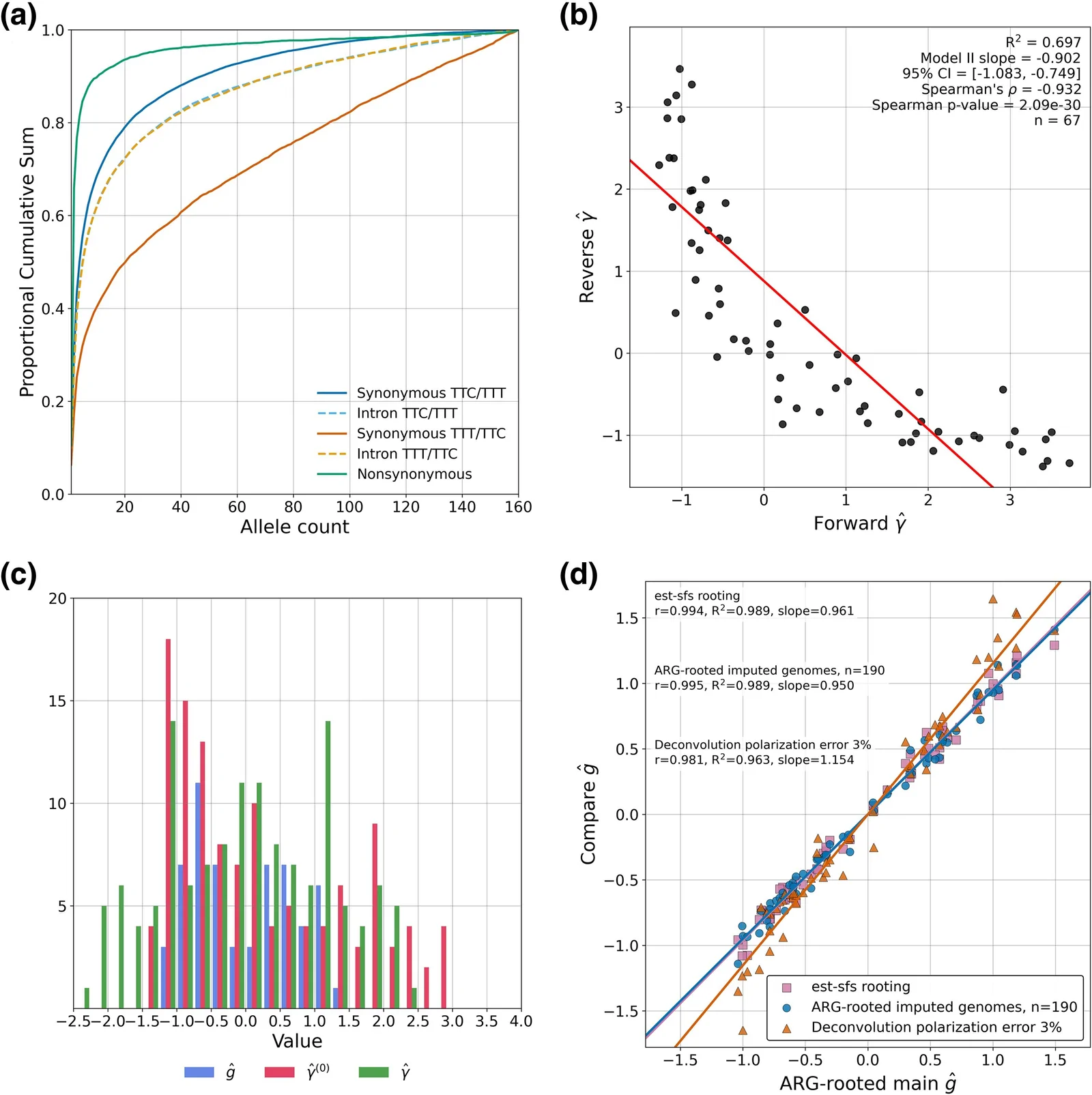
a) Cumulative site frequency spectra, shown as proportional cumulative sums, for both directions of the phenylalanine synonymous codon pair TTC/TTT together with paired short-intron data and a nonsynonymous SFS for comparison. b) Correlation between estimates of forward and reverse gamma values for 67 randomly oriented reciprocal codon-pair comparisons. c) Histograms of codon fitness estimates $\left(\hat{g}\right)$, initial $2Ns\;\;({\hat{\gamma }}^{\left(0\right)})$ and final 2Ns estimates $\left(\hat{\gamma }\right)$. d) Comparison of alternative pipelines for generating codon fitness values, including est-sfs rooting, ARG-rooted imputed genomes with *n* = 190, and 3% deconvolution.

We take advantage of the commonality among instances of a class of synonymous variants by applying a new method for estimating the strength of selection for each of the 134 pairs of ancestral/descendant synonymous codon pairs that are one mutational step apart. Figure 1a shows an example of the cumulative site-frequency spectrum (SFS) for one ordered synonymous single-nucleotide polymorphism (SNP) pair for phenylalanine from the autosomes of aligned haploid genomes of a *Drosophila melanogaster* population from Zambia. Shown with each spectrum is a partner neutral spectrum from short intron SNPs of the same mutational class (i.e. C to T or T to C) and the same flanking base sequences. Also shown is the spectrum for nonsynonymous SNPs, to provide perspective. SNPs with deleterious derived alleles will have fewer high-frequency alleles (i.e. a cumulative curve above the neutral control), whereas SNPs with beneficial derived alleles will have more high-frequency alleles and a cumulative curve below the neutral control. In the case of both ordered changes for phenylalanine codons, Fig. 1a shows how the synonymous changes in one direction (TTC→TTT) are above the partner neutral control (i.e. consistent with negative selection) and changes in the other direction (TTT→TTC) are below the neutral control (i.e. consistent with positive selection).

Because the synonymous and matched intron spectra for each ordered codon pair should be affected similarly by demographic and other nonselective factors, including mutation and gene conversion, the strength of selection on the synonymous SNPs can be estimated from the ratio of selected to neutral site counts, particularly when selection is weak. This ratio-based approach also has the advantage of not relying on divergence data, codon frequencies, or prior assumptions about preferred codons (Hey and Pavinato 2025).

For n aligned genomes, the likelihood of the ratios for the two site frequency spectra, one for a specific synonymous codon change and a second for neutral sites from short introns (Parsch et al. 2010), is $\overset{n-1}{\underset{i=1}{\sum }}\;\mathrm{Log}(p(z_{i},\gamma ))$ (Hey and Pavinato 2025). Here $p(z_{i},\gamma )$ is the probability of $\;z_{i}$, the ratio of the count of synonymous codon sites with *i* derived alleles to neutral sites with *i* derived alleles, as a function of the strength of selection *γ*. As with virtually all likelihoods built upon a Poisson-random field model (Sawyer and Hartl 1992), this approach assumes that different sites segregate independently. The likelihood was maximized for each of the 134 pairs of site frequency spectra, yielding 134 codon-specific estimates of *γ*.

## Results

We used a panel of 197 haploid *D. melanogaster* genomes from Zambia (Lack et al. 2015). Variants were rooted using samples drawn from the posterior density of the ancestral recombination graph (ARG) for the Zambia genomes. Because of some missing data, random down-sampling to a sample size of 160 was used to generate the unfolded site SFS for each of the 134 ordered pairs of codons as well as a companion SFS from short introns for each ordered codon pair. We then applied the SFRatios method to the ratios of SFS counts for codon changes over intron changes (Hey and Pavinato 2025). This approach depends only on the ratios of frequencies of segregating variants and does not depend on any measures of divergence between species, nor any measure of codon usage or codon bias.

### Codon fitness estimates

For two alleles under weak selection with additive fitness, allele frequency dynamics are a function of the population selection coefficient, 2Ns (denoted hereafter as *γ*), where *N* is the effective population size and *s* is the selection coefficient. Under weak selection, the sign of 2Ns depends on whether the ancestral allele is favored or disfavored relative to the derived allele (Bulmer 1991). Thus each synonymous codon pair can provide two estimates of *γ*, with one ordered SFS providing a negative value and the reverse ordered SFS a positive value (Table S3).

Because both the forward and reverse values are estimated quantities with measurement uncertainty, conventional linear regression is not well suited for testing reciprocity in codon-pair selection estimates, as it assumes that the predictor variable is measured without error. Instead, we used a Model II regression with equal error variances, which treats error in both axes symmetrically and is therefore appropriate for testing the expected reciprocal relationship. Figure 1b shows a plot of the 67 pairs of initial *γ* estimates, denoted ${\hat{\gamma }}^{\left(0\right)}$. A Model II regression with equal error variances gave a slope of −0.902. Bootstrap inference using resamples of the 67 independent pairs gave a 95% confidence interval of [−1.083,−0.749], so the slope was not significantly different from the theoretical value of −1. The ordinary least-squares intercepts were positive on both axes (reverse on forward: 0.801; forward on reverse: 0.905). Our interpretation is that the data are consistent with the general form of the model, i.e. an approximately inverse linear relationship with slope near −1, but with a significant nonzero offset reflecting estimator bias.

To obtain internally consistent and less biased *γ* estimates, we constructed a least-squares estimator of a measure of codon fitness, *g*, for each of the 59 codons that can have synonymous mutations. The method assumes only that (i) the forward *γ* is the additive inverse of the reverse *γ*, and (ii) the mean fitness change across all codon changes for a given amino acid is zero. Final estimates of *γ*, denoted $\hat{\gamma }$, were then generated by taking the difference in $\hat{g}\;$ values for ordered pairs of codons. The distributions of $\hat{g}\;$ and initial ${\hat{\gamma }}^{\left(0\right)}$ and final $\hat{\gamma }$ values are shown in Fig. 1c (numerical values are in Tables S3, S4 and S5). Values of $\hat{\gamma }$ range from −2.30 to 2.30 with mean 95% confidence interval of 0.82, with 66 of the 134 ordered codon pairs having $\hat{\gamma }$ value between −1 and 1, the interval of values often considered to be *effectively* neutral.

Several variations on the *g* estimation pipeline were evaluated, including: (i) using an alternative rooting protocol that used outgroup data as well as the SFS; (ii) using a larger sample (n=190) of imputed genomes; and (iii) using a deconvolution algorithm to generate data that mimics a 3% ancestral polarization error rate. All three of these variations generated $\hat{g}\;$ values similar to the original values (Table S6; Fig. 1d).

We were unable to identify any pattern of covariation with $\hat{\gamma }$ among several features of amino acids. The mean codon fitness does not seem to covary with the number of codons or the number of tRNA genes for an amino acid in the *D. melanogaster* genome, or properties of the R-group, including charge, size, or hydrophobicity (Table S7).

### Predicted codon frequencies

**Figure 2:**
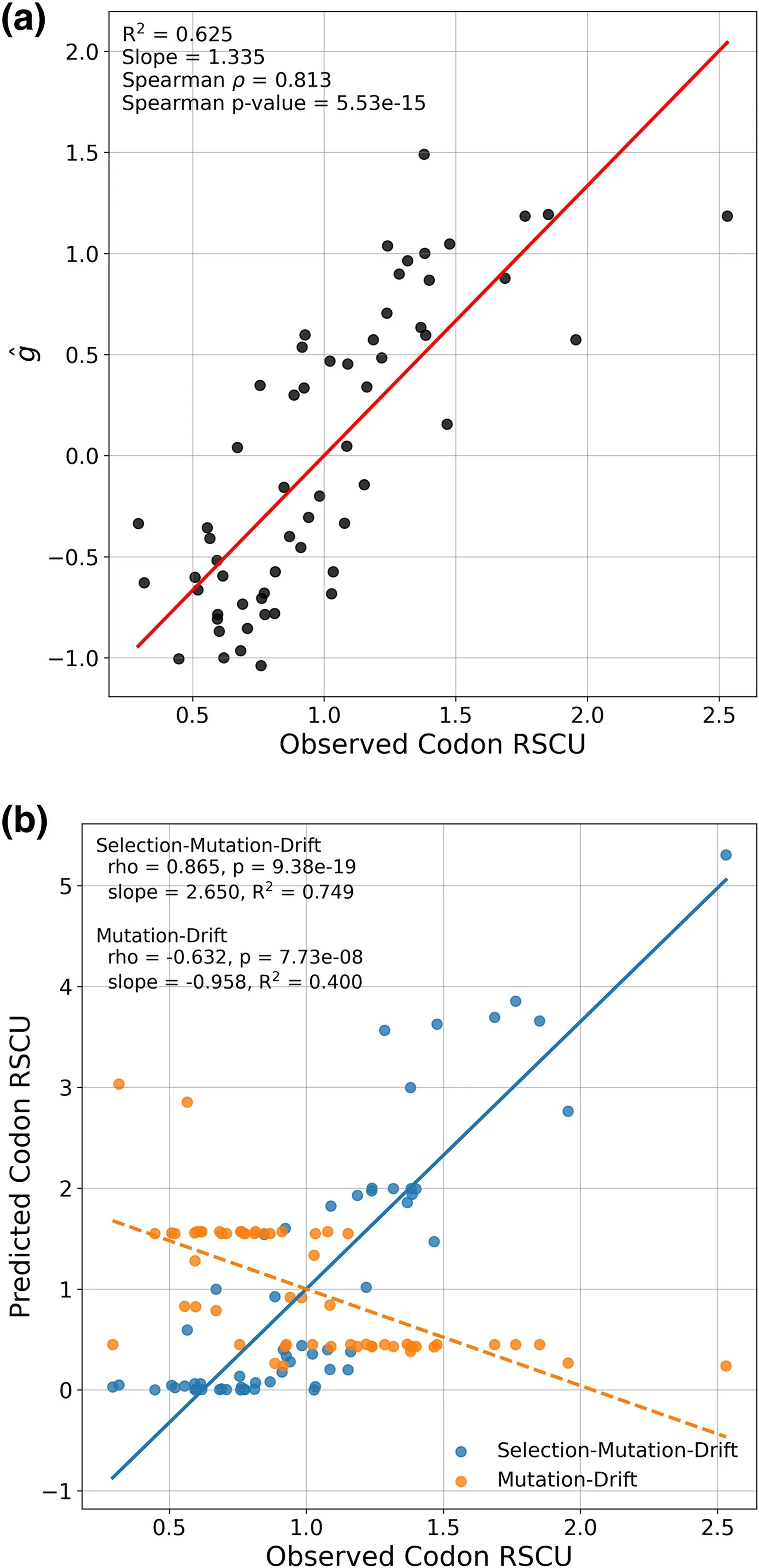
a) Comparison between codon fitness $\left(\hat{g}\right)$ and the RSCU (scaled codon frequencies) observed in the *D. melanogaster* reference genome. b) Association between the observed and the expected normalized codon frequencies under a selection-mutation-drift model and under a mutation-drift-only model.

We then examined the correlation between $\hat{g}\;$ and relative synonymous codon usage (RSCU), a measure of codon frequency that scales by the redundancy of the corresponding amino acid. For codon *i* with observed frequency $p_{i}$ for an amino acid with *k* codons $\mathrm{RSC}U_{i}=k*p_{i}$ (Sharp et al. 1986), so that an RSCU of 1 indicates usage equal to the synonymous expectation, whereas values above or below 1 indicate relative enrichment or depletion. This normalization facilitates comparisons among codons belonging to amino acids with different levels of codon redundancy. Although $\hat{g}\;$ is based on only relative frequencies of synonymous variants, it is strongly correlated with codon frequency (Fig. 2a).

To further examine the associations with codon frequency, we predicted codon frequencies under both a selection-mutation-drift model (based on $\hat{\gamma }$ values as well as estimated relative mutation rates) and a mutation-drift model that includes only the relative mutation rates. The model with selection returned RSCU values that were strongly correlated with the observed values in the *D. melanogaster* reference genome; however, the slope of the relationship (2.65) means that the $\hat{\gamma }$ values predict a stronger departure from equal codon usage than actually observed (Fig. 2b). In principle, this could be due to $\hat{\gamma }$ values having higher absolute values than true 2Ns values. However, the selection-mutation-drift model assumes that all three forces are in equilibrium, whereas the estimates of *γ* are relatively immune to the equilibrium assumption (Hey and Pavinato 2025).

The model with selection predicted actual codon frequencies far better than the model with only mutation. In fact, there is a negative association between the predicted and observed RSCU values for the model without selection. This is not unexpected because of the known mismatch between the base values of codons that occur at high frequency and the known mutation spectrum in *D. melanogaster*. Most of the more common codons end in G or C (Vicario et al. 2007), whereas the estimated base-pair mutation matrix has higher rates from GC pairs to AT pairs rather than the reverse (Assaf et al. 2017). If mutation alone were the primary driver of synonymous codon frequencies, then we would expect common codons to typically end in A or T.

### Codon fitness and gene expression

As in many species, the most common phenotype that has been associated with variation in codon usage in *D. melanogaster* is gene expression, with selection apparently acting more strongly in genes that are highly expressed (Sharp and Li 1986; Duret and Mouchiroud 1999). But gene expression is a crude measure that, while correlated with various measures of codon usage in *D. melanogaster*, may obscure important effects in individual tissues (Payne and Alvarez-Ponce 2019; Allen et al. 2022) or life stages (Vicario et al. 2008). Codon usage in *D. melanogaster* has also been shown to have effects at many steps between transcription and protein, including translation speed (Comeron et al. 1999; Zhao et al. 2017; Wu et al. 2024), translational accuracy (Akashi 1994; Wu et al. 2024), modulation of co-translational folding of proteins (Fu et al. 2016; Zhao et al. 2017), and altering the steady-state mRNA levels through codon-dependent mRNA decay (Burow et al. 2018). The connection between codon usage and gene expression could arise either from gene-specific effects of codon choice on the expression or function of the gene in which it occurs, or from broader cellular costs of slow or inaccurate translation that are experienced more strongly for genes translated at higher rates (Shah and Gilchrist 2011).

Regardless of the driving mechanism, the central hypothesis underlying associations between gene expression and codon usage is that the selective impact of a synonymous codon change increases with the expression level of the gene containing that codon. Thus, codons that have a negative effect on gene expression, relative to other codons for the same amino acid, will tend to occur less frequently in genes that are highly expressed. A corollary is that codons with stronger selective effects should show larger expression-associated shifts in usage frequency: favored codons should increase in frequency in highly expressed genes, whereas disfavored codons should decrease, with weaker-effect codons showing smaller changes. We examined this hypothesis using the codon fitness estimates, $\hat{g}$, by fitting gene-level multinomial models of synonymous codon usage as a function of gene expression summed over life stages (Hu et al. 2017). We used the log of gene expression, rather than raw values, because the summed expression values were extremely right-skewed, spanning more than five orders of magnitude (median of 361 fragments per kilobase of transcript per million mapped reads [FPKM] and a maximum of 136,001).

**Figure 3:**
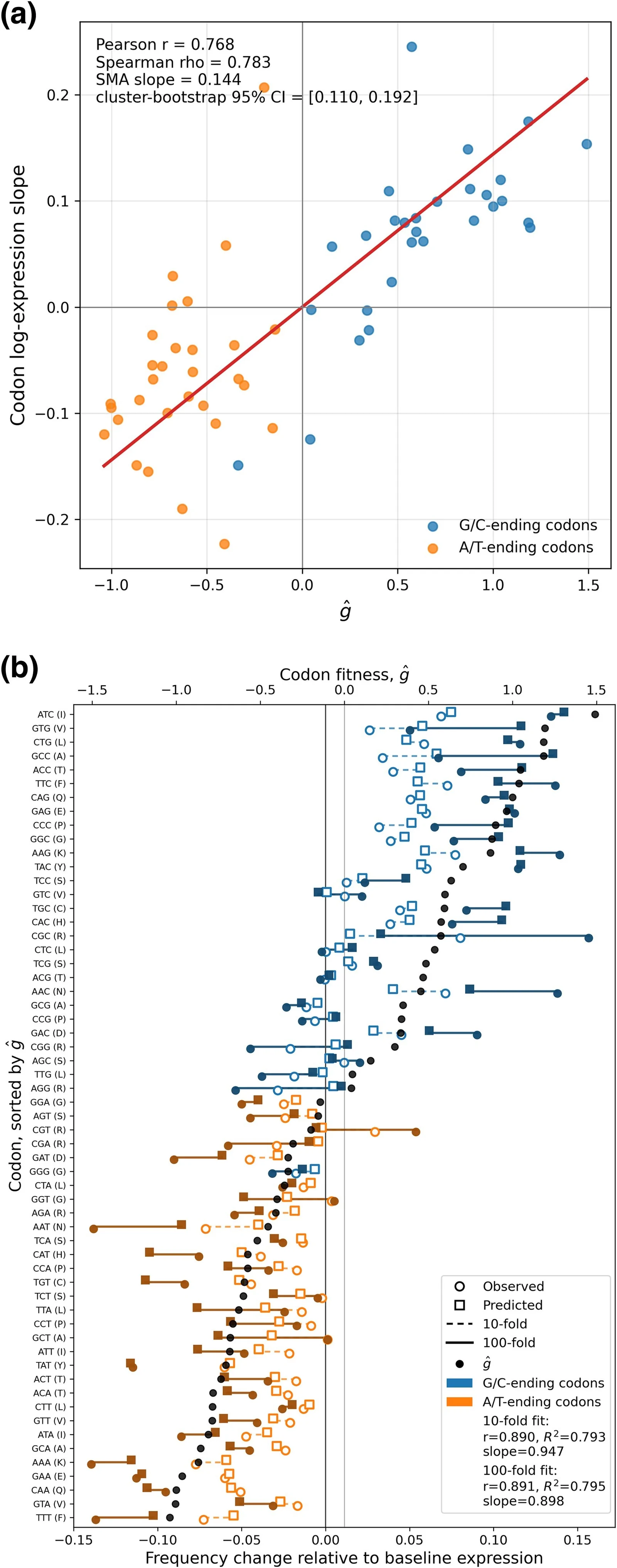
a) Association between codon fitness and gene-expression effects on synonymous codon usage. b) Observed and model-predicted codon frequency changes with increasing gene expression. Codons are sorted by the codon fitness scale shown on the upper *x* axis.

The model that includes codon-specific expression slopes fit much better than a null model with expression-independent codon intercepts only (ΔAIC = 44,203.1; likelihood-ratio statistic = 44,285.1, df = 41, $P<10^{-300}$), showing that RSCU changes systematically with gene expression. The estimated codon-specific expression slopes were strongly positively associated with $\hat{g}\;$ (Pearson r=0.768, $R^{2}=0.590$, Spearman ρ=0.783;  Fig. 3a). Figure 3a also shows that the G/C-ending codons are concentrated toward higher $\hat{g}$ values and more positive expression slopes, consistent with their increased use in highly expressed genes.

Given that selection is stronger in more highly expressed genes, we developed an expression-scaled mutation-selection-drift model in which each gene is assigned a selection scale, *λ* determined by that gene's expression level. Increasing the selection scale for gene $r\;(i.e.\;\;\lambda_{r})$ increases the effect of codon fitness on predicted codon frequencies, which in turn increases the expected frequency of codons with positive $\hat{g}$ values and decreases the expected frequency of codons with negative $\hat{g}$ values. The fitted expression coefficient was positive (β=0.098), indicating that the selection scale increases with gene expression. A 10-fold increase in the measure of expression (FPKM) was predicted to increase $\lambda_{r}\;$ by a factor of 1.141, corresponding to an approximately 14.1% increase in the strength of codon selection. The 200-replicate bootstrap confidence interval was 1.130 to 1.153, supporting a consistent positive effect of increasing gene expression on the strength with which codon fitness differences shape synonymous codon usage.

Comparing high- and low-expression genes, $\lambda_{r}$ has a mean of 0.735 among the 10% of genes with the highest expression, and a mean of 0.513 among the 10% with the lowest expression, corresponding to a ratio of 1.43. Thus, the fitted model predicts that the codon-selection scale, or equivalently the strength with which codon fitness differences affect synonymous codon usage, is about 43% stronger in the most highly expressed genes compared to the least highly expressed genes.

Figure 3b compares, across the range of estimated codon fitness values, the observed expression-associated changes in codon frequency with the changes predicted by the expression-scaled mutation-selection-drift model. The comparison shows that the model captures the main qualitative pattern: codons with greater absolute values of $\hat{g}$ have greater predicted and observed frequency changes. For both 10-fold and 100-fold changes in expression level, the predicted and observed changes are highly correlated across the 134 codon changes ($R^{2}$ = 0.793 and 0.795, respectively), consistent with much of the expression-associated change in codon usage being aligned with the independently estimated codon fitness scale.

### Codon fitness and codon usage bias

**Figure 4:**
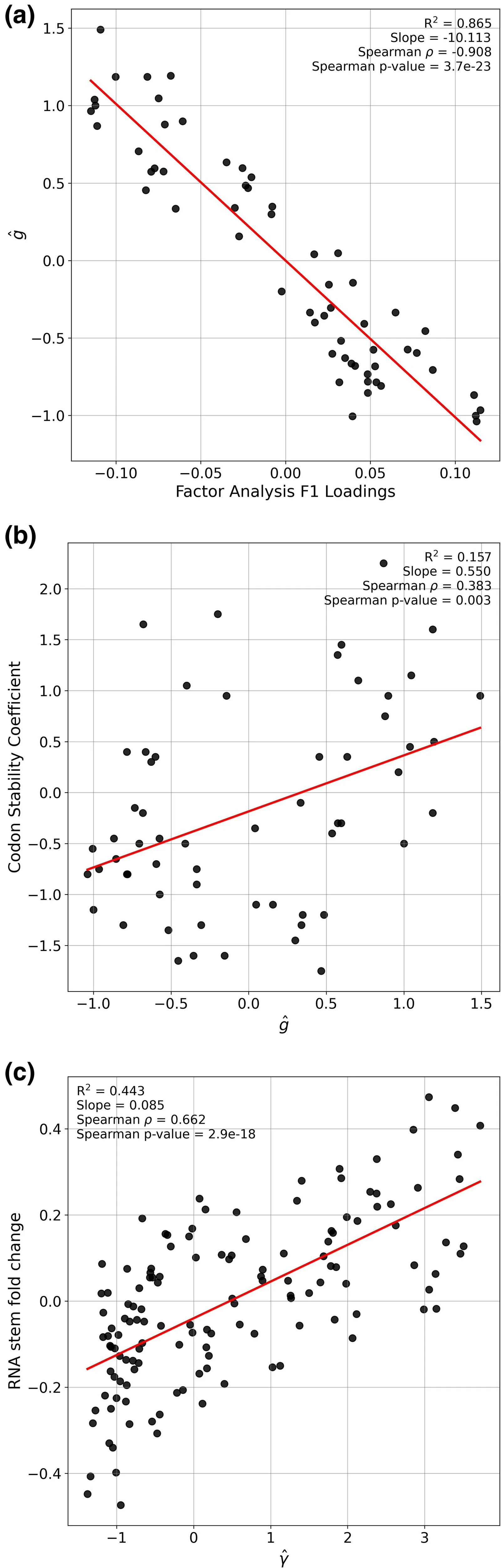
a) Correlation between factor analysis F1 loadings and $\hat{g}$. b) Correlation of codon stability coefficient and $\hat{g}$. c) Correlation of model-fitted $\hat{\gamma }$ and mean stem/loop fold change for each ordered codon pair.

One way to capture the effect of factors that act similarly on codons across a spectrum of genes is to identify patterns of covariation in codon usage bias among genes. If gene expression is the primary driver, for example, then high-expression genes will share a subset of the “favored” codons across the 18 amino acids with multiple codons. This pattern of covariation can be captured directly with a multivariate analysis of codon frequency covariation (Hey and Kliman 2002; Rahman et al. 2021). We conducted a factor analysis of codon-frequency covariation across the genes of *D. melanogaster* (similar to principal components analysis and correspondence analysis). The first factor from this analysis reflects the largest axis of covariation in codon frequencies across genes, and if this is driven by natural selection, then we expect to see a strong association between the Factor 1 loadings on individual codons and our measure of codon fitness. Figure 4a shows the very strong ($R^{2}=0.865$) association of the first factor from a factor analysis of codon frequency covariation with $\hat{g}$ (the sign of the association, negative in this case, is arbitrary).

### Codon fitness and mRNA stability

Synonymous codon changes can alter transcript half-life if they alter mRNA secondary structure, which in turn affects mRNA stability (Mauger et al. 2019). At the codon level, it has been shown in *D. melanogaster* that different codons are associated with different impacts on mRNA stability (Burow et al. 2018). The authors measured decay rates for nearly 8,000 mRNAs in *D. melanogaster* embryos and calculated the codon stabilization coefficients (CSC), a measure of the correlation between codon usage and transcript stability (Presnyak et al. 2015). They found CSC to be positively correlated with a measure of tRNA gene copy number. When we examined the association between $\hat{g}$ and CSC, we found a statistically significant positive correlation between CSC and codon fitness (Spearman ρ=0.383, p=0.003,  Fig. 4b).

We further examined the hypothesis that selection on synonymous codon usage is partly mediated through mRNA folding by estimating secondary structure and the average impact of each type of synonymous codon change on mRNA secondary structure. For each of over 12,000 mRNA sequences, we estimated secondary structure stem and loop assignments for each base position, and then for each of the 59 synonymous codons determined the mean frequency of occurrence in estimated stem portions of mRNA secondary structures. Then, for each of the 134 types of synonymous SNP, we calculated the change in relative stem proportion as the difference in relative stem proportion for the derived codon minus that of the ancestral codon. Model-fitted selection coefficients $\left(\hat{\gamma }\right)$ show a highly significant correlation with the change in the stem enrichment score of the codon (Pearson r=0.666, $R^{2}=0.443$, $P=1.7\times 10^{-18}$; Fig. 4c), consistent with RNA stability being an important component of codon usage's impact on gene expression.

## Discussion

Synonymous codon variation has long been thought to be under selection in *D. melanogaster* (Shields et al. 1988; Sharp and Li 1989; Moriyama and Hartl 1993). However, the strength of this selection has been uncertain, with reports that selection in *D. melanogaster* is undetectable (McVean and Vieira 2001) or that selection is very weak (|γ|<1) (Akashi 1995), or even quite strong (Machado et al. 2020).

To better examine the strength of selection, we applied a new approach that relies only on ratios of frequencies of segregating sites for candidate selected sites (i.e. synonymous codon changes) and a neutral control (SNPs in short introns) (Hey and Pavinato 2025). For each of 134 pairs of synonymous codons (ancestral/derived) for which the codons are one base apart, we obtained an initial set of estimates of *γ*. We then exploit a key corollary of the hypothesis of weak selection, that a change of synonymous codon A to B will have a selection coefficient that is the additive complement of the change of B to A. When we fit this model to the initial $\hat{\gamma }\;$ values, we obtained estimates of codon-specific fitnesses (i.e. $\hat{g}$), which we then used in turn to generate a final set of model-fitted $\hat{\gamma }$ values.

The model-fitted estimates fall in a narrow range $|\hat{\gamma }|<2.30$. Although the strength of selection is quite modest, it is nevertheless strong enough to shape the site frequency spectra and to have a large effect on codon frequencies. In particular, the most common codons are GC-ending, and yet these are expected to be at lower frequencies because of the mutational pressure in favor of AT-ending codons. The fitness estimates predicted codon frequencies quite well under a simple selection-mutation-drift model, whereas predictions based on mutation rates alone fail to predict codon frequencies. We also observed a strong correlation between estimates of *g* and the first factor from a factor analysis of the codon frequency covariation that is a dominant feature of codon usage bias in *D. melanogaster*. Finally, we detect a clear signal that selection on codon usage acts in the direction of stabilizing mRNA secondary structure. These observations are consistent with a general model in which gene-level selection on codon usage drives patterns of genome-wide variation in codon usage.

The codon fitness estimates reported here are aggregate measures that do not reflect the strength of selection on codon usage in individual genes. Given that the codon-specific associations with gene expression strongly covary with codon fitnesses (Fig. 3a), it appears that gene expression is a main driver of codon usage variation, as many other studies have shown. To address the question that is raised by these results—i.e. “how does the strength of selection change when gene expression changes?”—we fit an expression-scaled mutation-selection-drift model in which gene expression modifies the scale of selection acting on synonymous codon fitness differences. This model predicted that a 10-fold increase in FPKM increases the strength of selection on synonymous codon usage by approximately 14%, and that highly expressed genes (top 10%) experience selection on synonymous codon usage only about 43% stronger than genes in the bottom 10%. Thus, although gene expression significantly modulates the selective consequences of synonymous codon usage, the fitted effect is modest over the observed range of expression values. Even doubling the codon fitness estimates reported here would not yield any |2Ns| values greater than 10, and a doubling of the fitted selection scale would require expression values outside the range observed in these data.

The modest fitted dependence of selection strength on expression level is not inconsistent with the strong association between codon usage and gene expression. In the mutation-selection-drift framework, even small differences in codon fitness can produce substantial differences in equilibrium codon frequencies. Consistent with this, selection explains much more of the observed synonymous codon usage pattern than mutation alone (Fig. 2b), and the estimated codon fitnesses covary strongly with the dominant axis of codon-frequency variation among genes (Fig. 4a). Thus, the finding that $|\hat{\gamma }|\leq \;2.30$ indicates that relatively weak selection is sufficient to generate strong codon-usage structure among *Drosophila* genes, while expression acts mainly as a modest modifier of that selection.

In the fitted expression-scaled model, a gene with average log-expression has a selection-scaling factor of (λ=0.63), nearly identical to the expression-independent estimate from the constant model. Thus, if all genes were expressed at the same level, the model would reduce to an approximately constant scaling of the codon fitness estimates, with expression no longer contributing gene-to-gene variation in selection strength. The similarity between the expression-independent selection scale and the selection scale estimated for genes with average expression suggests that much of the codon-usage signal is not attributable solely to variation in expression level. Instead, expression appears to act as a modest modifier of a broader, expression-independent component of selection on synonymous codon usage.

After the initial reports of selection on codon usage in *D. melanogaster* based on associations with gene expression (Shields et al. 1988) and the negative correlation between synonymous substitution rates and codon usage bias (Sharp and Li 1989), subsequent studies reached differing conclusions about the strength and prevalence of selection. Some found little or no evidence of widespread selection (McVean and Vieira 2001; Singh et al. 2007), whereas others inferred very weak selection, in some cases weaker than estimated here (Akashi 1995, 1996; Nielsen et al. 2007). More recent studies have instead inferred that a substantial fraction of synonymous codons may be under relatively strong selection, with some estimates exceeding 2Ns>10 (Lawrie et al. 2013; Machado et al. 2020). One thing that these diverse studies, including those reporting very weak or no selection and those reporting strong selection, share that is different from the present study is a reliance on species divergence data and counts of synonymous positions that are invariant within species.

Divergence data integrate evolutionary processes over much longer timescales than polymorphism data and are therefore exposed to additional sources of variation, including alignment error, changes in mutation bias, changes in base composition, and shifts over time in which codons are favored by selection (Akashi et al. 2006; Bauer DuMont et al. 2009; Singh et al. 2009; Selberg et al. 2025). Invariant codon positions can be highly informative in a fully specified mutation-selection model, but they greatly outnumber polymorphic sites and can therefore exert strong leverage on parameter estimates. Small errors in defining, filtering, counting, or assigning invariant sites to codon classes, or in modeling the corresponding mutation opportunities, could in principle bias estimates either downward or upward, depending on how the errors interact with the assumed mutation-selection model.

Our approach does not depend on between species divergence, or on overall codon abundance, or relative codon frequencies, and therefore does not rely on prior designations of “preferred” or “unpreferred.” Moreover, because they are based on ratios of frequencies that are largely insensitive to demographic history—particularly when selection is weak (Hey and Pavinato 2025)—they reflect recent selective forces in the population. Finally, the estimates appear robust to ancestral/derived polarization errors given the similarity of results from two unrelated polarization procedures, and the modest impact on estimates of a 3% polarization error rate applied using a deconvolution algorithm.

## Methods

### Polymorphism data

We extracted synonymous and short intron SNPs from whole-genome sequencing data for the four autosomal chromosome arms of 197 haploid embryo genomes from Zambia (Lack et al. 2015). All sequence data were downloaded from the Drosophila Genome Nexus (DGN, https://www.johnpool.net/genomes.html). We first ran the “masking package” available at DGN to mask identical-by-descent or admixture tracts when present in one or many genomes. Using a custom pipeline that uses the snp-sites program (Page et al. 2016), the multi-alignment FASTA file of the 197 genomes was converted to a VCF file. We used GATK LiftoverVcf to shift the SNP coordinate positions to Dromel 6 (dos Santos et al. 2015) and annotated the VCF files with SnpEff (Cingolani et al. 2012) as done previously (Hey and Pavinato 2025). We rooted the VCF file using two methods, one that used outgroup data together with polymorphism data and a second method that made use of the ARG for the set of aligned Zambia genomes (see below). To examine the effect of using all genomes by imputing the missing data, we used Beagle v.5.5 (Browning and Browning 2007) to build a VCF file with a sample size of 190 genomes. The imputed VCF file was also used for ARG estimation, which requires that no data be missing at SNP sites (see below). To help ensure that the control SNPs were selectively neutral and as closely matched as possible for local mutational context, we focused on short introns (Parsch et al. 2010) (less than 86 base pairs, with the first and last 8 bases excluded). For each ordered codon pair, the intron SFS was assembled by identifying, for each synonymous SNP, a short intron SNP that had the same reference allele and flanking bases (Machado et al. 2020; Hey and Pavinato 2025).

### Ancestral base estimation

For the study of selection, unfolded site frequency spectra (i.e. from a count of 1 up to a count of n−1) are preferable because they preserve the full sample frequency information for each allele, whereas folding the spectrum collapses complementary frequency classes (i.e. counts for allele count *i* are combined with counts for allele count n−i) (Fu 1995; Nielsen et al. 2005; Tataru et al. 2017). To estimate which of the two bases at a biallelic SNP is ancestral and which is derived, we applied two methods: one that uses both outgroup and polymorphism information, and one that relies solely on estimates of the ARG.

To polarize *D. melanogaster* SNPs using outgroup and polymorphism data, we used the est-sfs program (Keightley and Jackson 2018). We compiled a version of est-sfs that uses five ordered outgroups with the multi-rate R6 mutation model (Keightley and Jackson 2018). A whole-genome multiple sequence alignment was built using Cactus v1.3.0 and the cactus-hal2maf script (Armstrong et al. 2020) for *D. melanogaster* and outgroup species in increasing order of distance: *Drosophila simulans*, *Drosophila yakuba*, *Drosophila subpulchrella*, *Drosophila ananassae*, and *Drosophila miranda*. Soft-masked reference genome assemblies were obtained from NCBI RefSeq (Table S1). If est-sfs reported a base as being ancestral with probability greater than or equal to 0.999, then that base was set to ancestral. In all other cases, the reference base in the VCF file was treated as the ancestral base.

One of the difficulties using outgroup data is that many of the SNPs are not represented in all of the species in the multispecies alignment. An alternative rooting protocol is to jointly estimate the genealogical history at each SNP and thus rely upon the patterns of allele frequencies and linkage among nearby SNPs to estimate the timing and direction of each mutation (Liang and Dutheil 2026). We used the SINGER program (Deng et al. 2021) to draw 10 samples from the ARG posterior distribution of the imputed Zambia genome set. The imputed VCF file was first divided into two Megabase regions, and SINGER was run on each with options of effective population size of $1.1\times 10^{6}$ (Sheehan and Song 2016), mutation rate $2\times 10^{-9}$ per base pair (Keightley et al. 2014) and genetic maps from Comeron et al. (2012).

The estimates of 2Ns for the two rooting methods were very similar (Fig. 1d). Because the ARG-based method evaluated all SNPs in the imputed data set and gave the most complete polarization, the ARG-rooted analysis was used as the primary analysis throughout the study.

### Assembly of selected and neutral SFSs for each ordered codon pair

Site frequency spectra were built for each of the 134 ordered (ancestral to derived) synonymous codon pairs from the polarized SNPs in the Zambia VCF files. Because of some missing data, SFSs were built to a common sample size of 160 by random down-sampling with replacement.

For each ordered codon pair SFS, a neutral partner SFS of the same total allele count was built by identifying, for each synonymous SNP, a short intron SNP that had the same reference allele and the same flanking bases (Machado et al. 2020; Hey and Pavinato 2025).

### Simulations of deconvoluted polarization errors

To approximate an error-free SFS from data with possible mispolarization (i.e. incorrect identification of the ancestral allele), we assumed a uniform mispolarization rate of 0.03 and treated each mirror pair of forward and reverse bins (e.g. TTT→TTC and TTC→TTT; derived-allele counts 1 to 159) as a 2 × 2 linear mixing system between the forward (F) and reverse (R) spectra.

Assuming a mispolarization rate (*r*), the observed forward and reverse spectra are modeled as mixtures of the unseen true spectra. For N=160 genomes, i=1,…,N−1 and j=N−i: $F_{\mathrm{obs}}[i]=(1-r)F_{\mathrm{true}}[i]+rR_{\mathrm{true}}[j]$ and $R_{\mathrm{obs}}[j]=(1-r)$  $R_{\mathrm{true}}[j]+rF_{\mathrm{true}}[i]$ (and symmetrically for $R_{\mathrm{obs}}[i],F_{\mathrm{obs}}[j]$). We inverted this 2×2 system independently for each mirror pair to obtain deconvolved expectations. The deconvolved forward expectation is $F_{\exp}[i]=(\left(1-r)F_{\mathrm{obs}}[i]-rR_{\mathrm{obs}}[j\right])/(1-2r)$, with analogous expressions for $R_{\exp}[j],\;R_{\exp}$, and $F_{\exp}[j]$. Small negative expectations were truncated to zero. We generated simulated deconvolved spectra by multinomial sampling from these expected values for all 134 pairs of SFSs, while preserving the original number of polymorphic sites in each SFS.

### Fitting a codon fitness model

We consider a model in which selection on the alleles at a synonymous SNP depends only on the codon identities of the segregating alleles, without dominance and without any epistatic interactions with other parts of the genome. Furthermore, we suppose that synonymous SNPs are segregating just two alleles (sites with more than two alleles were excluded), in which case the intensity of selection on a SNP can be modeled as the difference in the fitness value of the two segregating codons. Under this model, for an amino acid with 2-fold redundancy, the selection coefficient associated with a change in one direction will be the negative of the selection coefficient of the change in the reverse direction. For example, consider all SNPs for the 2-fold amino acid tyrosine. When the ancestral state is the codon TAC, and the derived state is TAT, we would expect under our simple model that the SFS from all TAC > TAT SNPs could be modeled as being shaped by a selection coefficient *γ*, while the SFS for those SNPs that are TAT > TAC could be modeled using the additive inverse of *γ*, i.e. −γ. To examine the prediction that *γ* estimates from both ordered SFSs for a codon pair should be close to additive inverses of each other, forward and reverse pairs were regressed against each other under a model II regression (data associated with both *x* and *y* axes are subject to error), assuming 67 independent points.

For an amino acid encoded by *k* synonymous codons, there are k(k−1) possible ordered codon changes. When k=2, the two ordered changes are reciprocal, so the pairwise estimates directly describe the fitness difference between the two codons. When k>2, however, there are more ordered codon-pair estimates than codons, and the pairwise estimates are not independent if they arise from an underlying set of *k* codon fitnesses. We therefore summarized the ordered pair estimates by fitting a codon-fitness model in which each codon i has a fitness value $g_{i}$, and the selection coefficient for a mutation from codon *i* to *j* is $\gamma_{i,j}=g_{j}-g_{i}$. Under this model, the selection coefficient for any ordered codon change is determined by the difference between the fitness of the derived codon and the fitness of the ancestral codon. Adding the same constant to all $g_{i}$ values within an amino-acid family leaves every difference $g_{j}-g_{i}$ unchanged, so the absolute scale of *g* is not identifiable. Thus, only relative codon fitnesses can be estimated: one codon can be assigned an arbitrary reference value, or equivalently the $g_{i}$ values can be centered within each amino-acid family.

To fit this model, we populate a *k* by *k* matrix with ${\hat{\gamma }}^{\left(0\right)}$ values, set the first codon to have g=0, and then estimated the remaining k−1  *g* values by minimizing the squared deviations between the matrix of ${\hat{\gamma }}^{\left(0\right)}\;$ values and the corresponding matrix of $g_{j}-g_{i}$ values:


$$\overset{k}{\underset{i=1}{\sum }}\;\overset{k}{\underset{\;j=1,j\neq i}{\sum }}\;({\hat{\gamma }}_{i,j}^{\left(0\right)}-(g_{j}-g_{i}){)}^{2}.$$

Finally, we subtract from each of the *k* fitness values (including that with g=0) the mean $\bar{g}$ so that the final estimated values have a mean of zero. As the selection intensities depend only on the difference in *g* values, this rescaling retains the basic arithmetic relationship between $\hat{g}$ and ${\hat{\gamma }}^{\left(0\right)}\;$ values. Imposing a mean of zero, as assumed under the model, will also act to remove the directional bias observed in the initial ${\hat{\gamma }}^{\left(0\right)}$ values.

Bootstrap confidence intervals for $\hat{\gamma }\;$ and $\hat{g}\;$ values were obtained by sampling paired synonymous and short-intron SNP entries with replacement to build 120 data sets, each with 134 pairs of synonymous and short-intron SFSs. *γ* and *g* estimates were obtained for each bootstrap data set just as for the actual data, and the 95% confidence intervals were estimated from the ordered bootstrap values for each array of $\hat{\gamma }\;$ and $\hat{g}$.

To assess whether the $\hat{g}$ values provide a statistically significant fit to the $\hat{\gamma }$ values, for a given amino acid with k>2 synonymous codons, we calculated the correlation between the matrix of initial ${\hat{\gamma }}^{\left(0\right)}$ values and the matrix of $\hat{\gamma }\;$ values. If the $k\times (k-1)\;{\hat{\gamma }}^{\left(0\right)}$ values are indeed related by an underlying set of *k* codon fitness values, then the correlation between k×(k−1) pairs of ${\hat{\gamma }}^{\left(0\right)}$ and $\hat{\gamma }\;$ values should be higher than for a randomly shuffled set of ${\hat{\gamma }}^{\left(0\right)}$ values (i.e. randomly assigned to codons). For amino acids with more than two codons, this correlation was compared to 1,000 simulated correlations obtained by fitting g values for 1,000 matrices of randomly shuffled $\hat{\gamma }$ values. The proportion of simulations that provide a higher correlation amounts to a *p* value for the hypothesis that the ${\hat{\gamma }}^{\left(0\right)}\;$ values are associated with each other as specified under the model. For all amino acids with more than two codons the observed correlation was the greater than or equal to either all, or nearly all of 1,000 simulations (Table S7).

### Expected codon frequencies

A number of studies have been conducted to estimate the base pair mutation rates in *D. melanogaster*, and if we take the overall mean of these estimates (Assaf et al. 2017), we can build a matrix of the relative mutation rates between all possible pairs of bases. This can then be used to predict codon frequencies under an equilibrium model of only mutation, as well as a model with $\hat{\gamma }$ values. Under a Wright–Fisher model with transition rate $q_{i,j}\;$ from codon *i* to codon *j*, the equilibrium frequency of codon *i*, $\pi_{i}$, is subject to detailed balance such that $\pi_{i}q_{i,j}=\pi_{j}q_{\;j,i}$ at equilibrium, which gives the relation


$$\pi_{i\;}/\pi_{j}=q_{\;j,i}/q_{i,j}.$$

Then, given a set of transition rates and the constraint that $\overset{k}{\underset{i=1}{\sum }}\;\pi_{i}=1$, the system of equations of the same form as expression (1) for all pairs of codons for an amino acid, can be reduced to a solution for the equilibrium frequencies.

In a model with weak selection the transition rate from codon *i* to codon *j* is $u_{i,j}\times e^{2N(s_{j}-s_{i})}$ (Ewens 2004) so that $\pi_{i}/\pi_{j}=\frac{u_{\;j,i}}{u_{i,j}}e^{2(2Ns_{i}-2Ns_{j})}$. Thus given a set of mutation rate estimates and 2Ns estimates, we can estimate the equilibrium codon frequencies under a selection-mutation-drift equilibrium model. The same framework, with 2Ns values set to a constant can be used to predict equilibrium codon frequencies under a mutation-drift model, i.e. without selection.

### Gene expression data

We obtained all life-stage data for all 12,690 Drosophila genes in the DGET database (Hu et al. 2017) and summed FPKM values across life stages to get an overall gene expression value. Genes were ranked by expression and divided into 104 equally sized bins. Codon frequencies were measured in the lowest and highest bins. For a measure of “difference in codon frequency,” we first measured relative difference as the absolute difference in mean frequency among genes in each of the highest and lowest bins, divided by the mean of the frequencies in the two bins. The final measure was this relative difference minus the average of relative differences among the 59 synonymous codons.

### Estimating the association between gene expression and codon fitness

To test whether synonymous codon selection varies with gene expression, we assessed the dependence of gene-level codon counts on gene expression estimates and then asked how estimates of codon-specific slopes covary with codon fitness estimates $\hat{g}$. For each gene, codon counts were obtained from *D. melanogaster* coding sequences from Ensembl (Dyer et al. 2025). Expression data summaries, FPKM values, and codon counts by gene are in Table S9. For each gene, a standardized measure of expression was calculated from the mean and standard deviation among 12,656 genes with FPKM values and codon counts. For gene *r*, let $E_{r}=\log\;(\mathrm{FPK}M_{r}+1)$. Standardized expression was then calculated as $X_{r}=(E_{r}-u)/s$, where *u* and *s* are the mean and standard deviation of $E_{r}$ across genes.

We first fit gene-level multinomial models of synonymous codon usage to estimate codon-specific associations with gene expression. For amino acid a, let $S_{a}$ be the set of synonymous codons for that amino acid, let $n_{ir}$ be the observed count of codon in gene *r*, $n_{ar}$ be the vector of $n_{ir}$ values for amino acid *a*, and let $N_{ar}=\underset{i\in S_{a}}{\sum }\;n_{ir}$. Conditional on $N_{ar}$, the probability of the counts for the codons for amino acid a in gene *r* is


$$P_{ar}(n_{ar})=\frac{N_{ar}!}{\prod_{i\in S_{a}}\;n_{ir}!}\underset{i\in S_{a}}{\prod }\;q_{ir}^{n_{ir}}\;.$$

The codon probabilities were modeled with a multinomial logit parameterization,


$$q_{ir}=\frac{exp(\mu_{i}+b_{i}X_{r})}{\sum_{\;j\in S_{a}}\;exp(\mu_{j}+b_{j}X_{r})},$$

where $X_{r}$ is the standardized gene expression, $\mu_{i}$ is the expression-independent codon intercept, and $b_{i}$ is the codon-specific expression slope. The multinomial-logit models were fit using one reference codon per amino-acid family for identifiability. After fitting, intercepts and expression slopes were transformed to a centered parameterization by subtracting the mean fitted value within each synonymous codon family, so that the centered slopes summed to zero within each family. These centered slopes were used for comparisons with codon fitness estimates.

This model estimates, for each codon, whether its relative usage within its synonymous codon family increases or decreases with gene expression. The likelihood is


$$L(M,B)=\underset{a}{\prod }\;\underset{r}{\prod }\;P_{ar}(n_{ar}),$$

where M is the vector of codon-specific intercepts and B is the vector of codon-specific expression slopes. Likelihoods were maximized numerically; the multinomial-logit models were fit with L-BFGS-B using analytic gradients, and the expression-scaled model was fit by direct optimization over *α* and β. Codon-specific intercepts and expression slopes are reported in Table S10.

We compared codon-specific expression slopes with codon fitness estimates using correlation and standardized major-axis regression (Table S11). Confidence intervals for the standardized major-axis slope were obtained by cluster bootstrap resampling of amino-acid families, which preserves dependence among synonymous codons belonging to the same amino acid.

To assess whether including gene expression improved the fit of the model, we compared the expression-dependent full model to a null model that included expression-independent codon intercepts only. The likelihood for the null model used codon probabilities for which the logit parameterization used only the expression-independent codon intercept


$$q_{ir}^{\mathrm{null}}=\frac{\exp\;(\mu_{i})}{\sum_{\;j\in S_{a}}\;\exp\;(\mu_{j})}.$$

### Predicting the effect of gene expression on the strength of codon selection

We fit an explicit expression-scaled mutation-selection-drift model. This analysis differs from the preceding expression-slope analysis in that it used the mutation-only equilibrium codon frequencies, $p^{\mathrm{mut}}$, and the codon fitness estimates, $\hat{g}$, as fixed inputs, then measured the dependence of the strength of selection on gene expression. For codon i and amino acid *a*, the predicted probability that the codon takes value in gene *r* was


$$p_{ir}=\frac{\;p_{i}^{\mathrm{mut}}\exp\;(2\lambda_{r}{\hat{g}}_{i})}{\sum_{\left(\;j\in S_{a}\right)}\;p_{j}^{\mathrm{mut}}\exp\;(2\lambda_{r}{\hat{g}}_{j})},$$

where $S_{a}\;$ is the set of codons for *a* and $\lambda_{r}$ is the gene-specific selection scale. Larger values of $\lambda_{r}$ amplify the effect of codon fitness such that codons with positive ${\hat{g}}_{i}\;$ are predicted to increase in frequency as gene expression goes up, whereas codons with negative ${\hat{g}}_{i}\;$ are predicted to decrease in frequency.

The gene-specific selection scale was modeled as a log-linear function of gene expression, $\lambda_{r}=\exp(\alpha +\beta X_{r}).$ The parameter α is the fitted intercept, the baseline log selection scale of a gene with average standardized expression. The parameter *β* is the effect of expression on selection scale. Thus each gene has its own value of $\lambda_{r}$, but these values are determined by the shared fitted parameters *α* and *β*, together with the gene's observed expression value $X_{r}$. The likelihood was constructed from multinomial probabilities across genes and amino-acid families,


$$L(\alpha ,\beta )=\underset{r}{\prod }\;\underset{a}{\prod }\;\left[\frac{N_{ar}!}{\prod_{i\in S_{a}}\;n_{ir}!}\underset{i\in S_{a}}{\prod }\;p_{ir}^{n_{ir}}\right].$$

Because expression was modeled as standardized log(FPKM+1), the fitted coefficient *β* gives the change in log selection scale per one standard deviation of log expression. We therefore summarized the expression effect as the fold-change in $\lambda_{r\;}$ expected for a 10-fold increase in FPKM, calculated as $\exp\;\left(\frac{\beta \log\;(10)}{SD[\log\;(\mathrm{FPKM}+1)]}\right).$ Confidence intervals for this fold-change were obtained from 200 bootstrap replicates in which genes were resampled with replacement and, within each resampled gene and amino-acid family, synonymous codon counts were resampled from the observed count vector conditional on the total number of codons for that amino acid. Parameter estimates, expression-effect estimates, and bootstrap confidence intervals for the expression-scaled mutation-selection-drift model are reported in Table S12.

### Factor analysis

Codon frequencies were recorded for each of 59 synonymous codons for 12,905 genes in the *D. melanogaster* reference genome. For each amino acid with multiple codons, these frequencies summed to 1. A conventional factor analysis was conducted of the codon frequencies using the scikit-learn module (Kramer 2016) in python and the first factor (F1) was taken as a measure of codon frequency covariation across genes (Hey and Kliman 2002).

### RNA secondary structure analysis

Secondary structure was estimated for 12,761 mRNA sequences using the ViennaRNA package (Lorenz et al. 2011). For each codon, we estimated an average impact on RNA secondary structure as follows. For each of over 12,000 mRNA sequences, we estimated secondary structure stem and loop assignments for each base position, and then for each of the 59 synonymous codons determined the mean proportion of stem and loop occurrence in these estimated secondary structures. We then assigned to each codon a relative stem representation as its mean stem representation divided by the mean of stem representation of all codons (and similarly for relative loop representation). Then, for each of the 134 synonymous changes, we calculated a stem enrichment score as the difference in relative stem representation, i.e. the relative stem representation for the derived codon minus that for the ancestral codon.

## Supplementary Material

msag199_Supplementary_Data

## Data Availability

Analysis scripts are available under the MIT License at https://github.com/jodyhey/codon_fitness and are archived on Zenodo at https://doi.org/10.5281/zenodo.21537449.
